# Relationship Between Maximal Left Ventricular Wall Thickness and Sudden Cardiac Death in Childhood Onset Hypertrophic Cardiomyopathy

**DOI:** 10.1161/CIRCEP.121.010075

**Published:** 2022-05-02

**Authors:** Gabrielle Norrish, Tao Ding, Ella Field, Elena Cervi, Lidia Ziółkowska, Iacopo Olivotto, Diala Khraiche, Giuseppe Limongelli, Aris Anastasakis, Robert Weintraub, Elena Biagini, Luca Ragni, Terrence Prendiville, Sophie Duignan, Karen McLeod, Maria Ilina, Adrián Fernández, Chiara Marrone, Regina Bökenkamp, Anwar Baban, Peter Kubus, Piers E.F. Daubeney, Georgia Sarquella-Brugada, Sergi Cesar, Sabine Klaassen, Tiina H. Ojala, Vinay Bhole, Constancio Medrano, Orhan Uzun, Elspeth Brown, Ferran Gran, Gianfranco Sinagra, Francisco J. Castro, Graham Stuart, Gabriele Vignati, Hirokuni Yamazawa, Roberto Barriales-Villa, Luis Garcia-Guereta, Satish Adwani, Katie Linter, Tara Bharucha, Pablo Garcia-Pavia, Ana Siles, Torsten B. Rasmussen, Margherita Calcagnino, Caroline B. Jones, Hans De Wilde, Toru Kubo, Tiziana Felice, Anca Popoiu, Jens Mogensen, Sujeev Mathur, Fernando Centeno, Zdenka Reinhardt, Sylvie Schouvey, Costas O’Mahony, Rumana Z. Omar, Perry M. Elliott, Juan Pablo Kaski

**Affiliations:** Centre for Inherited Cardiovascular Diseases, Great Ormond Street Hospital, London, United Kingdom (G.N., E.F., E.C., J.P.K.).; Institute of Cardiovascular Sciences (G.N., C.O., P.M.E., J.P.K.), University College London, United Kingdom.; Department of Statistical Science (T.D., R.Z.O.), University College London, United Kingdom.; The Children’s Memorial Health Institute, Warsaw, Poland (L.Z.).; Careggi University Hopsital, Florence, Italy (I.O.).; Necker–Enfants Malades Hospital, Paris, France (D.K.).; Monaldi Hospital, Naples, Italy (G.L.).; Onassis Cardiac Surgery Center, Athens, Greece (A.A.).; Royal Children’s Hospital, Melbourne, Australia (R.W.).; Cardiology Unit, S. Orsola-Malpighi Hospital, IRCCS Azienda Ospedalierao-Universitaria di Bologna, Italy (E.B., L.R.).; Our Lady’s Children’s Hospital, Dublin, Ireland (T.P., S.D.).; Royal Hospital for Children, Glasgow, United Kingdom (K.M., M.I.).; Fundación Favaloro University Hospital, Buenos Aires, Argentina (A.F.).; Papa Giovanni XXIII Hospital, Bergamo (C.M.).; Fondazione Toscana G. Monasterio, Massa-Pisa, Italy (C.M.).; Leiden University Medical Center, the Netherlands (R.B.).; Bambino Gesu Hospital, Rome, Italy (A.B.).; University Hospital Motol, Prague, Czech Republic (P.K.).; Royal Brompton and Harefield NHS Trust, London, United Kingdom (P.E.F.D.).; Sant Joan de Deu, Barcelona, Spain (G.S.-B., S.C.).; Department of Pediatric Cardiology (S.K.), Charite–Universitatsmedizin Berlin, Germany.; Experimental and Clinical Research Center, a joint cooperation between the Charité Medical Faculty and the Max-Delbrück-Center for Molecular Medicine (S.K.), Charite–Universitatsmedizin Berlin, Germany.; German Centre for Cardiovascular Research, Partner Site Berlin, Germany (S.K.).; Department of Pediatric Cardiology, Pediatric Research Center, New Children’s Hospital, University of Helsinki, Finland (T.H.O.).; Birmingham Children’s Hospital, United Kingdom (V.B.).; Hospital General Universitario Gregorio Marañón, Madrid, Spain (C.M.).; University Hospital of Wales, Cardiff (O.U.).; Leeds General Infirmary, United Kingdom (E.B.).; Val d’Hebron University Hospital, Barcelona, Spain (F.G.).; Heart Muscle Disease Registry Trieste, University of Trieste, Italy (G.S.).; University Hospital Virgen de la Arrixaca, Murcia, Spain (F.J.C.).; Bristol Royal Hospital for Children, United Kingdom (G.S.).; Niguarda Hospital, Milan, Italy (G.V.).; Department of Pediatrics, Faculty of Medicine and Graduate School of Medicine, Hokkaido University Hospital, Sapporo, Japan (H.Y.).; Complexo Hospitalario Universitario A Coruna, INIBIC, CIBERCV, Spain (R.B.-V.).; University Hospital La Paz, Madrid, Spain (L.G.-G.).; John Radcliffe Hospital, Oxford (S.A.).; Glenfield Hospital, Leicester (K.L.).; Southampton General Hospital, Southampton, United Kingdom (T.B.).; Hospital Universitario Puerta de Hierro Majadahonda, Madrid, Spain (P.G.-P., A.S.).; Aarhus University Hospital, Denmark (T.B.R.).; Fondazione IRCCS Ca’ Granda Ospedale Maggiore Policlinico Milano, Dept di Medicina Interna, UOC Cardiologica, Milano, Italy (M.C.).; Alder Hey Children’s Hospital, Liverpool, United Kingdom (C.B.J.).; Ghent University Hospital, Belgium (H.D.W.).; Kochi Medical School Hospital, Japan (T.K.).; Mater Dei Hospital, Malta (T.F.).; Department of Pediatrics, University of Medicine and Pharmacy “Victor Babes” Timisoara, Children’s Hospital ‘Louis Turcanu,’ Romania (A.P.).; Aalborg University Hospital, Denmark (J.M.).; Evelina Children’s Hospital, London, United Kingdom (S.M.).; Rio Hortega University Hospital, Valladolid, Spain (F.C.).; The Freeman Hospital, Newcastle, United Kingdom (Z.R.).; Hospital Saint Joseph, Marseille, France (S.S.).; St Bartholomew’s Centre for Inherited Cardiovascular Diseases, St Bartholomew’s Hospital, West Smithfield, London, United Kingdom (C.O., P.M.E.).

**Keywords:** adult, child, death, sudden, humans, hypertrophic cardiomyopathy

## Abstract

**Background:**

Maximal left ventricular wall thickness (MLVWT) is a risk factor for sudden cardiac death (SCD) in hypertrophic cardiomyopathy (HCM). In adults, the severity of left ventricular hypertrophy has a nonlinear relationship with SCD, but it is not known whether the same complex relationship is seen in childhood. The aim of this study was to describe the relationship between left ventricular hypertrophy and SCD risk in a large international pediatric HCM cohort.

**Methods:**

The study cohort comprised 1075 children (mean age, 10.2 years [±4.4]) diagnosed with HCM (1–16 years) from the International Paediatric Hypertrophic Cardiomyopathy Consortium. Anonymized, noninvasive clinical data were collected from baseline evaluation and follow-up, and 5-year estimated SCD risk was calculated (HCM Risk-Kids).

**Results:**

MLVWT *Z* score was <10 in 598 (58.1%), ≥10 to <20 in 334 (31.1%), and ≥20 in 143 (13.3%). Higher MLVWT *Z* scores were associated with heart failure symptoms, unexplained syncope, left ventricular outflow tract obstruction, left atrial dilatation, and nonsustained ventricular tachycardia. One hundred twenty-two patients (71.3%) with MLVWT *Z* score ≥20 had coexisting risk factors for SCD. Over a median follow-up of 4.9 years (interquartile range, 2.3–9.3), 115 (10.7%) had an SCD event. Freedom from SCD event at 5 years for those with MLVWT *Z* scores <10, ≥10 to <20, and ≥20 was 95.6%, 87.4%, and 86.0, respectively. The estimated SCD risk at 5 years had a nonlinear, inverted U-shaped relationship with MLVWT *Z* score, peaking at *Z* score +23. The presence of coexisting risk factors had a summative effect on risk.

**Conclusions:**

In children with HCM, an inverted U-shaped relationship exists between left ventricular hypertrophy and estimated SCD risk. The presence of additional risk factors has a summative effect on risk. While MLVWT is important for risk stratification, it should not be used either as a binary variable or in isolation to guide implantable cardioverter defibrillator implantation decisions in children with HCM.

**What Is Known?**

- Maximal left ventricular wall thickness is a risk factor for sudden cardiac death in childhood hypertrophic cardiomyopathy.
- Maximal left ventricular wall thickness is included in current risk stratification guidelines.

**What the Study Adds**

- An inverted U-shaped relationship exists between left ventricular hypertrophy and estimated sudden cardiac death risk in childhood hypertrophic cardiomyopathy.
- The presence of additional risk factors has a summative effect on risk.

**See Editorial by Kohli et al**

Sudden cardiac death (SCD) is the most common mode of death in childhood hypertrophic cardiomyopathy (HCM), occurring at an annual rate of 1% to 2%.^1–4^ Maximal left ventricular wall thickness (MLVWT) is a recognized risk factor for SCD in childhood^5–11^ and adult-onset disease,^12–14^ and it is designated as a major clinical risk factor in current risk stratification guidelines.^15–17^ Current pediatric risk stratification recommendations suggest using a threshold for MLVWT to guide implantable cardioverter defibrillator (ICD) implantation decisions,^15,17^ and although they differ in the threshold for defining extreme hypertrophy (eg, MLVWT ≥30 mm,^15^ *Z* score ≥6,^15,17^ *Z* score ≥20^17^), the implication is that increasing MLVWT is associated with increasing risk. Recently published pediatric-specific risk models include continuous measures of left ventricular hypertrophy (LVH) as risk factors,^18,19^ but the relationship between LVH and SCD is incompletely understood. In adults with HCM, LVH has an inverted U-shaped relationship with the risk of SCD,^20^ but it is not known whether this same complex, nonlinear relationship exists in childhood HCM. The aim of this study was to describe the relationship between LVH and observed and predicted SCD risk in a large pediatric cohort.

## Methods

### Study Population and Data Collection

The study cohort was derived from the International Paediatric Hypertrophic Cardiomyopathy Consortium—a retrospective, multicentre, longitudinal study consisting of 1198 children diagnosed with nonsyndromic HCM aged 1 to 16 years from 49 participating centers previously used to develop (n=1024) and validate (n=174) the pediatric HCM risk prediction model (HCM Risk-Kids).^19^ For this analysis, 123 patients with incomplete MLVWT data were excluded, resulting in a final study cohort of 1075 patients. HCM was defined as MLVWT ≥2 SDs above body surface area^21^–corrected population mean (*Z* score ≥+2) that could not be explained by abnormal loading conditions.^15^ *Z* scores were calculated using normative data from the Pediatric Heart Network Normal Echocardiogram Database.^22^ Patients with a history of previous ventricular fibrillation or sustained ventricular tachycardia, inborn errors of metabolism, RASopathy syndrome, or neuromuscular disease were excluded from the analysis.

Anonymized, noninvasive clinical data were collected from baseline evaluation and follow-up, including heart failure symptoms (New York Heart Association or Ross functional classification^23^), family history, resting and ambulatory ECG, and transthoracic echocardiography (2-dimensional, Doppler, and color). The presence or absence of the following additional clinical risk factors at the time of baseline evaluation was recorded: nonsustained ventricular tachycardia (NSVT; defined as ≥3 consecutive ventricular beats at a rate of >120 beats per minute lasting <30 seconds on ambulatory ECG recordings^15^), unexplained syncope, family history of SCD (defined as a sudden death in a first-degree relative under the age of 40 years or sudden death at any age in a first-degree relative with HCM^15^), left ventricular outflow tract (LVOT) obstruction (defined as an instantaneous peak Doppler LVOT pressure gradient ≥30 mm Hg at rest^15^), and body surface area–corrected left atrial diameter. Severity of LVH was divided into groups (MLVWT *Z* score <10, MLVWT *Z* score ≥10 to <20, and MLVWT *Z* score ≥20). Genetic testing information was not routinely collected. Data were collected independently at each participating center, and data integrity is guaranteed by each author.

### Outcomes

Primary study outcomes were major arrhythmic cardiac event (MACE), defined as SCD or an equivalent event (aborted cardiac arrest, appropriate ICD therapy, or sustained ventricular tachycardia associated with hemodynamic compromise). Secondary outcomes were all-cause mortality or cardiac transplantation. Outcomes were determined by the treating cardiologist at each participating center.

### Statistical Analysis

Continuous variables are described as mean (±SD) or median (25th to 75th centile) as appropriate, with 3 group comparisons conducted using ANOVA or Wilcoxon rank-sum, respectively. Categorical variables were compared using the χ^2^ test. The correlation between MLVWT and continuous variables was assessed using Pearson or Spearman rank correlation as appropriate. Follow-up time was calculated from the date of baseline evaluation to the date of reaching the study primary end point, death from another cause, or date of most recent evaluation. Estimates of survival by MLVWT group were obtained using the Kaplan-Meier product limit method. MLVWT groups (MLVWT *Z* score <10, MLVWT *Z* score ≥10 to <20, and MLVWT *Z* score ≥20) were prespecified before analysis. The association of continuous MLVWT *Z* score with 5-year MACE and mortality was assessed in a univariate Cox proportional hazard model.

Patients with >3 HCM Risk-Kids predictor variables missing were excluded. Missing data were seen in 1, 2, or 3 predictor variables in 310 (28.8%), 108 (10.15%), and 12 (1.1%) patients, respectively. Data were assumed to be missing at random, and missing predictors were imputed using multiple imputations based on chained equations.^24^ The imputation model included the outcome, HCM Risk-Kids predictor variables, and an estimate of the cumulative hazard function. A total of 40 imputed data sets were created.

Follow-up was censored at 5 years, and the estimated 5-year risk of SCD was calculated for each patient using the previously published HCM Risk-Kids model.^19^

*P*(SCD at 5 years)=1−0.949437808^exp(prognostic index)^, where prognostic index=0.2171364×(MLVWT *Z* score−11.09)−0.0047562×(MLVWT *Z* score^2^−174.12)+0.130365×(left atrial diameter *Z* score−1.92)+0.429624×unexplained syncope+0.1861694×NSVT−0.0065555×(maximal LVOT gradient−21.8).

We have previously observed a nonlinear relationship between MLVWT *Z* score and 5-year SCD risk using a Cox regression model in the development cohort.^19^ The relationship between MLVWT *Z* score and 5-year SCD risk was examined graphically in a single imputed data set in those with and without coexisting dichotomous risk factors for SCD (NSVT, family history of SCD, and unexplained syncope) to show the effect on calculated risk estimates. A line of best fit was drawn using linear regression with a quadratic term for MLVWT *Z* score. The summative effect of multiple coexisting risk factors on estimated 5-year SCD risk estimates was examined graphically by setting continuous variables to their mean value for the study cohort and dichotomous variables to 0 (absence) or 1 (presence).

Statistical analysis was performed using the Stata statistical software (version 14).

### Ethics

This study conforms to the principles of the Declaration of Helsinki and Good Clinical Practice. Local ethical approval was given for each participating center with waiver of informed consent for retrospective, anonymized data. The data underlying this article cannot be shared publically as consent for dissemination of patient data was not obtained.

## Results

Mean age at diagnosis was 10.2 years (±4.4), and 716 (67.7%) were men. A family history of HCM or SCD was present in 583 (55.2%) and 133 (12.4%) patients, respectively.

### Baseline Clinical Phenotype

Mean MLVWT was 19.3 (±7.5) mm with a corresponding MLVWT *Z* score of 11.1 (±7.1). MLVWT *Z* score was <10 in 598 (58.1%), ≥10 to <20 in 334 (31.1%), and ≥20 in 143 (13.3%) patients (Figure S1). The baseline clinical characteristics by MLVWT *Z*-score group are described in the Table. Those with higher MLVWT *Z* scores were more likely to have heart failure symptoms, unexplained syncope, LVOT obstruction, left atrial dilatation, and NSVT. Age was not significantly associated with degree of LVH (Figure S1).

**Table.**
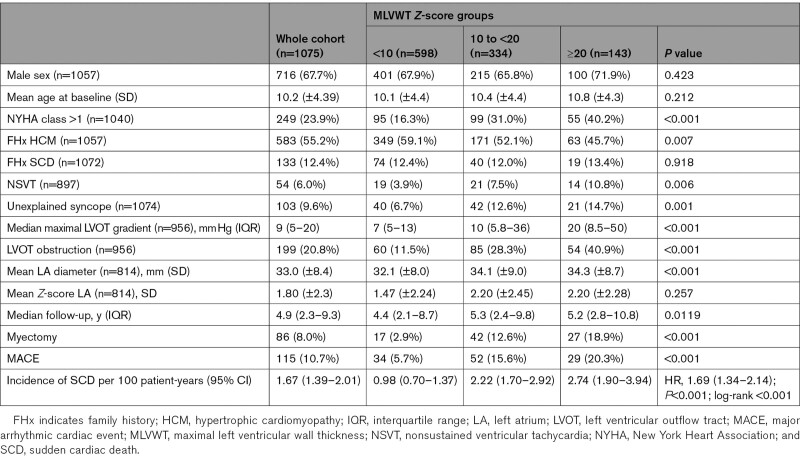
Summary of Baseline Characteristics by Severity of Left Ventricular Hypertrophy

### Relationship Between MLVWT and Other Clinical Risk Factors for SCD

**Figure 1.**
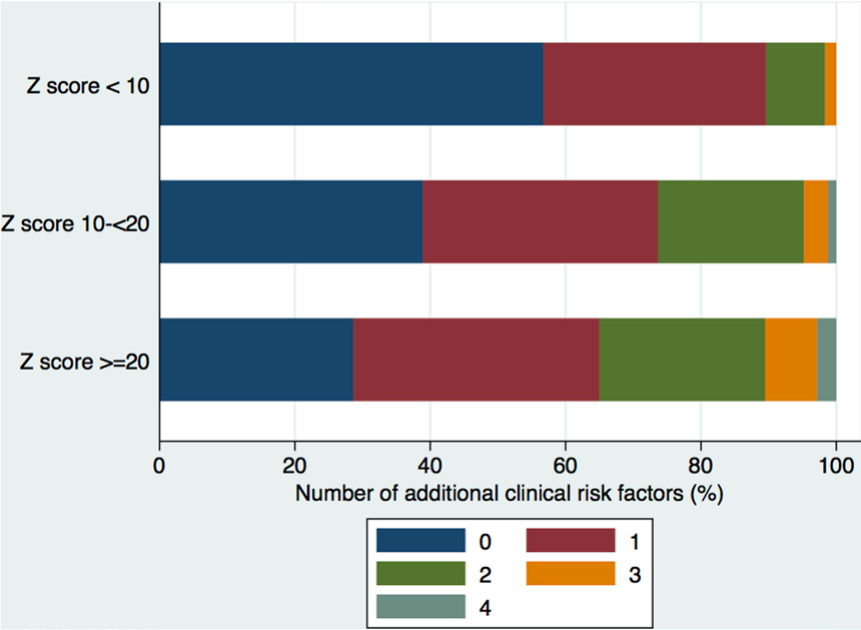
**The presence of additional clinical risk factors by severity of left ventricular hypertrophy.** Additional risk factors include unexplained syncope, nonsustained ventricular tachycardia, left atrial dilatation, and family history of sudden cardiac death.

One or more coexisting risk factors for SCD were present in 565 (52.6%) patients. Coexisting risk factors were more common in those with the most severe LVH (*Z* score <10 [n=259; 43.3%] versus *Z* score ≥10 to <20 [n=204; 61.1%] versus *Z* score ≥20 [n=102; 71.3%]; *P*<0.001; Figure 1). MLVWT correlated weakly with left atrial diameter *Z* score (R^2^, 0.028; *P*<0.001) and LVOT gradient (R^2^, 0.284; *P*<0.001).

### Relationship of MLVWT to MACE

**Figure 2.**
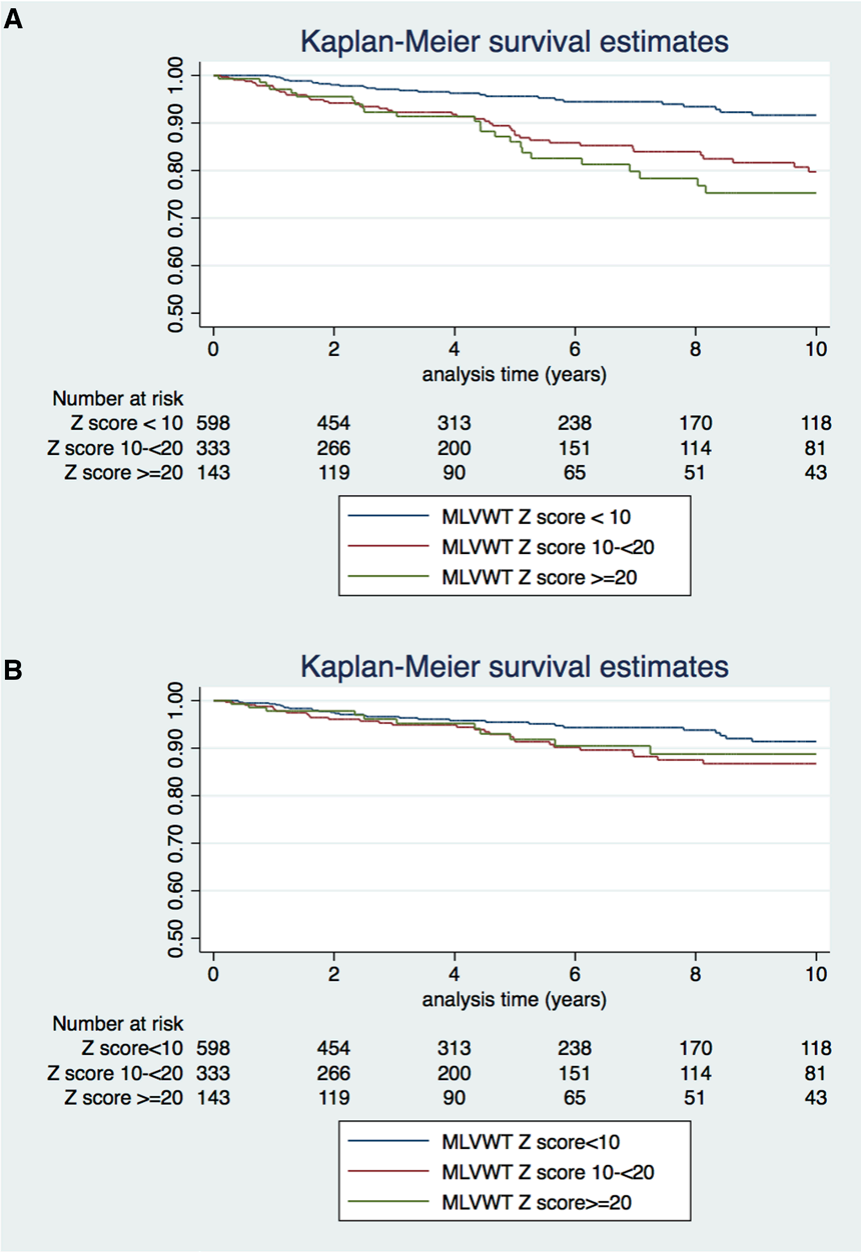
**Kaplan-Meier survival curves by degree of left ventricular hypertrophy. A**, major arrhythmic cardiac event or (**B**) all-cause mortality or cardiac transplantation. MLVWT indicates maximal left ventricular wall thickness.

One hundred and fifteen patients (10.7%) had an MACE (SCD [n=45; 39.1%]), resuscitated cardiac arrest (n=21; 18.3%), appropriate ICD therapy (n=38; 33.0%), or sustained ventricular tachycardia with hemodynamic compromise (n=11; 9.6%) with an overall incidence rate of 1.7/100 patient-years at risk (95% CI, 1.39–2.01). Freedom from MACE at 5 years for those with MLVWT *Z* score <10, MLVWT *Z* score ≥10 to <20, and MLVWT *Z* score ≥20 was 95.6% (95% CI, 93.2–97.2), 87.4% (95% CI, 82.6–91.0), and 86.0% (95% CI, 0.78–0.91), respectively (hazard ratio, 1.69 [95% CI, 1.34–2.15]; *P*<0.001; Figure 2A).

### Relationship Between MLVWT and HCM Risk-Kids Predicted Risk of SCD

**Figure 3.**
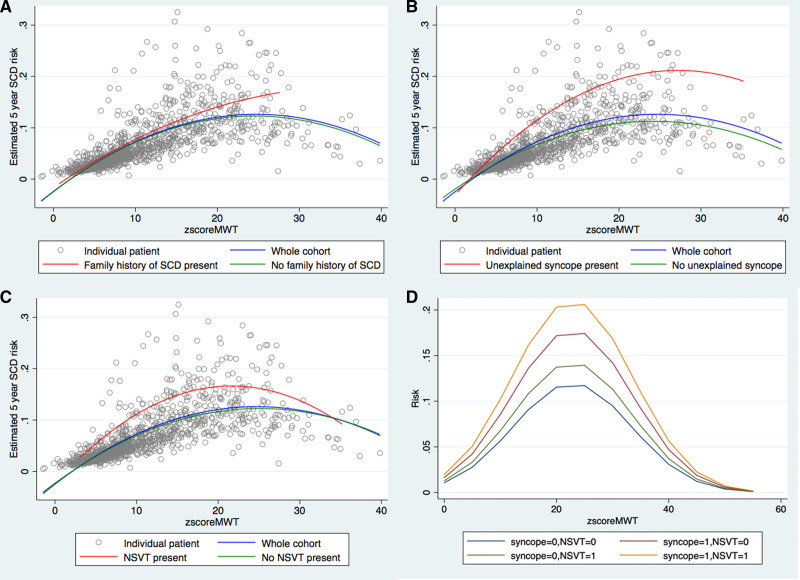
**Relationship of estimated 5-y risk of sudden cardiac death (SCD) to maximal wall thickness and the presence or absence of additional clinical risk factors. A**, The presence or absence of family history of SCD. **B**, The presence or absence of unexplained syncope. **C**, The presence or absence of nonsustained ventricular tachycardia (NSVT). **D**, All possible combinations of NSVT and unexplained syncope keeping continuous risk factors constant to the cohort mean: maximal left ventricular outflow tract gradient and left atrial diameter *Z* score. In all cases, the risk of SCD increases up to a point, and once a plateau is reached, the risk declines. MWT indicates maximal wall thickness.

In a single imputed data set, the estimated risk of SCD at 5 years as calculated by the HCM Risk-Kids model had a nonlinear, inverted, U-shaped relationship with MLVWT *Z* score. Estimated risk peaked at a *Z* score of +23; further increases in MLVWT were associated with an initial plateau and then fall in the estimated risk of SCD. The relationship between estimated SCD risk, MLVWT, and the presence or absence of other additional coexisting risk factors is shown in Figure 3.

### Relationship of MLVWT and Mortality

Over a median follow-up of 4.9 years (interquartile range, 2.3–9.3), 61 patients died (5.7%) and 29 (2.7%) underwent cardiac transplantation. Cause of death was SCD (n=45; 73.8%), heart failure (n=6; 9.8%), embolic event (n=1; 1.6%), other cardiovascular (n=3; 4.9%), noncardiovascular (n=3; 4.9%), or unknown (n=3; 4.9%). Overall incidence of death or transplantation was 1.2/100 patient-years at risk (95% CI, 0.96–1.48). Severity of LVH was not associated with an increased incidence of all-cause mortality or transplant (hazard ratio, 1.044 [95% CI, 0.786–1.386]; *P*=0.766; Figure 2B).

## Discussion

This study reports the association between LVH and SCD risk in a large, geographically diverse, multicenter cohort of childhood HCM. Severity of LVH was associated with the presence of other phenotypic features of severe disease. Although the incidence of an arrhythmic event was the highest for those with MLVWT *Z* score ≥20, an inverted U-shaped relationship exists between the degree of hypertrophy and estimated risk of SCD at 5 years meaning that, beyond a threshold, no further increase in risk was seen and instead the risk starts decreasing. The presence of additional risk factors had a summative effect on risk, which highlights the complex interaction between LVH and other risk factors for SCD and suggests that measures of LVH alone cannot be used to predict risk in childhood HCM.

### LVH in Childhood HCM

LVH is a prerequisite for the diagnosis of HCM,^15,16^ but its progression during childhood is incompletely understood. The traditional paradigm suggests that increases in LVH are more likely to be seen in adolescence,^25^ with the implication that younger children have phenotypically mild disease. We and others have recently shown that LVH can develop at any age in familial HCM, including infants and young children,^26,27^ and the most recent 2020 American Heart Association/American College of Cardiology guidelines no longer have a lower age limit for clinical screening.^17^ This large cohort of children aged between 1 and 16 years with HCM provides further evidence that the severity of LVH is highly variable and not dependent on age. Not surprisingly, patients with severe LVH were more likely to be symptomatic and have other clinical phenotypic features of severe disease, including LVOT obstruction and NSVT on ambulatory ECG monitoring.

### LVH and Risk Prediction in Childhood HCM

Measures of LVH are the most studied SCD risk factor in childhood HCM.^11^ An association between LVH and the risk of SCD has been reported frequently in childhood disease, and severe LVH is considered to be a major clinical risk factor.^5–11^ Historically, the evidence supporting individual risk factors in childhood disease has been limited by small patient numbers, but recent large pediatric series confirm the importance of LVH in risk stratification for pediatric HCM.^18,19,28^ A major limitation in the study of potential risk factors for SCD in childhood HCM has been an inconsistency in the definitions or measurements of echocardiographic parameters. In particular, the measures of LVH vary widely in published pediatric studies (including interventricular septal thickness, left ventricular posterior wall thickness, septal thickness:cavity ratio, body surface area–corrected measurements, and MLVWT),^11^ with the result that the best measure of hypertrophy for risk stratification in childhood disease is currently unknown. Until recently, guidelines recommended using a threshold of MLVWT of ≥30 mm or *Z* score ≥6 to guide ICD implantation decisions.^15,16^ The evidence for using this particular threshold is limited,^8,10^ and recent North American guidelines have suggested that a threshold of *Z* score ≥20 may be more appropriate.^17^ The interpretation of all *Z*-score thresholds is hampered by the use of different normative data for *Z*-score calculations, each of which yields different *Z* scores for the same individual, yet the implication is that risk increases in a linear fashion with increasing LVH. Challenging this view, we have shown that, although those with the highest MLVWT had the highest incidence of MACE, an inverted U-shaped relationship exists between the degree of LVH and estimated risk of SCD at 5 years. These findings suggest that, beyond a threshold, further increases in MLVWT are not associated with an incremental increase in predicted risk and instead the predicted risk may start decreasing. Of note, although risk peaked at a *Z* score of 23 in this study, the specific threshold will depend on which normative data are used, but the underlying shape of the relationship will be unchanged. This finding is in keeping with recent reports from other independent pediatric populations, which have described a nonlinear association between interventricular septal thickness and left ventricular posterior wall thickness *Z* scores and SCD risk, plateauing at a *Z* score of 20 using the Boston normative data.^18,29,30^ It is also in keeping with the results of a large adult HCM study (n=3673)^20^ and suggests that the relationship between LVH and MLVWT is similar in adult and pediatric patients. The mechanism underlying these observations is unknown, but possible explanations include competing causes of death in those with severe LVH or alternative molecular arrhythmogenic pathways in those with milder hypertrophy. Indeed, previous studies have described arrhythmic events occurring in patients with variants in the *Troponin T* gene who had extensive myocyte disarray despite minimal hypertrophy.^31,32^

### Role of Additional Risk Factors for SCD

Previous reports have described the coexistence of multiple risk factors for SCD in individual patients with HCM,^33,34^ and in agreement with this, almost 75% of patients with the most severe hypertrophy had ≥1 additional SCD risk factors. In the presence of a single additional risk factor, although the overall relationship between LVH and estimated risk was unchanged, an upward shift in the risk curve was observed. This study, therefore, shows that coexistence of risk factors has a summative effect on estimates of 5-year SCD risk. The overall effect on risk also appeared to differ subtly for different clinical risk factors. In the presence of NSVT, the risk curve was shifted upward and to the left meaning that the absolute MLVWT *Z*-score threshold for maximal SCD risk was lower. In contrast, family history of SCD, which is not included as a risk estimate in HCM Risk-Kids, appeared to have less effect on the risk estimate except for at the highest MLVWT *Z* scores. Family history of SCD has robust evidence to support its use in adult cohorts, but a previous meta-analysis found insufficient evidence to support its use in childhood.^11^ More recent large-scale population registry studies have also failed to find a significant association between family history and arrhythmic events,^18,28^ suggesting an important difference between adult and childhood-onset disease, which is currently unexplained. Possible explanations for the absence of an effect of family history in childhood disease difference include a higher prevalence of de novo variants, low proportion of sarcomeric disease in the included cohorts, or incomplete reporting of family history. The observed summative effect on risk remained in the presence of multiple coexisting risk factors. Current guidelines differ in their treatment of patients with single risk factors, but our findings suggest that while MLVWT is important for risk stratification, it should not be used either as a binary variable or in isolation to guide ICD implantation decisions in children with HCM.

### Limitations

This study is limited by problems inherent to longitudinal retrospective studies including missing or incomplete data. Previous studies have shown an association between the severity of LVH and long-term outcomes, including cardiovascular mortality, in childhood HCM.^5,6,18,19,28,35–37^ In this study, LVH was not associated with all-cause mortality or cardiac transplantation, but patients with severe LVH were more likely to experience disease-related morbidity including the need for left ventricular septal myectomy or arrhythmic events. This discrepancy could be explained by patients being censored when they reached the primary end point of MACE meaning that if they subsequently developed heart failure or underwent cardiac transplantation, this event would not be captured. The long-term morbidity and mortality associated with childhood HCM is, therefore, not accurately represented in this cohort. This study excluded patients with underlying syndromic or metabolic disease; however, as genetic testing data were not routinely collected for all patients, a small subset may have an undiagnosed underlying etiology. As genetic testing information was not systematically collected for the HCM Risk-Kids development cohort, it was not possible to investigate the role of genotype in disease phenotype or its interaction with other clinical risk factors in this study. The role of genotype in disease progression and risk stratification in childhood disease remains unclear. Recent data from the SHaRE registry (Sarcomeric Human Cardiomyopathy Registry),^38^ of over 1000 children with HCM, did not find a higher lifetime risk of arrhythmic events for genotype-positive patients, and inclusion of genotype in an alternative pediatric-specific risk model (PRiMaCY [Precision Medicine for Cardiomyopathy]^18^) did not significantly improve model predictions. Nonetheless, future multicenter collaborative studies are required to investigate the contribution of genotype in disease progression and risk stratification for childhood HCM.

### Conclusions

In a large cohort of children with HCM, severe LVH was associated with other phenotypic features of severe disease and coexists with additional risk factors for SCD. Although the risk of an arrhythmic event is the highest for those with more severe hypertrophy (MLVWT *Z* score ≥20), an inverted U-shaped relationship exists between the degree of hypertrophy and estimated risk of SCD at 5 years. This means that, beyond a threshold, further increases in hypertrophy are not associated with additional risk and predicted risk may start decreasing. The presence of additional risk factors had a summative effect on risk. This study, therefore, suggests that, while MLVWT is important for risk stratification, it should not be used either as a binary variable or in isolation to guide ICD implantation decisions in children with HCM.

## Article Information

### Sources of Funding

This work was supported by the British Heart Foundation (grant number FS/16/72/32270) to Drs Norrish and Kaski. E. Field and Dr Kaski are supported by Max’s Foundation and Great Ormond Street Hospital Children’s Charity. Dr Kaski is supported by a Medical Research Council Clinical–National Institute for Health Research (NIHR) Clinical Academic Research Partnership award. This work is (partly) funded by the NIHR Great Ormond Street Hospital Biomedical Research Centre. Dr Omar and T.D. work at University College London Hospitals/University College London that received a proportion of funding from the UK Department of Health National Institute for Health Research Biomedical Research Centres funding scheme. This work was financially supported by the Foundation for Paediatric Research, Finland (Dr Ojala). Dr Fernandez has received speaker’s fees from Sanofi-Genzyme.

### Disclosures

None.

### Supplemental Material

Figure S1

## Supplementary Material


