# Supplementary material for: Relationship Between Maximal Left Ventricular Wall Thickness and Sudden Cardiac Death in Childhood Onset Hypertrophic Cardiomyopathy
Source: Circ Arrhythm Electrophysiol. 2022 May 2;15(5):e010075. doi: 10.1161/CIRCEP.121.010075 (PMC7612749; doi:10.1161/CIRCEP.121.010075)
Supplement: Supplementary file 1 [file hae-15-e010075-s001.pdf]

SUPPLEMENTAL MATERIAL

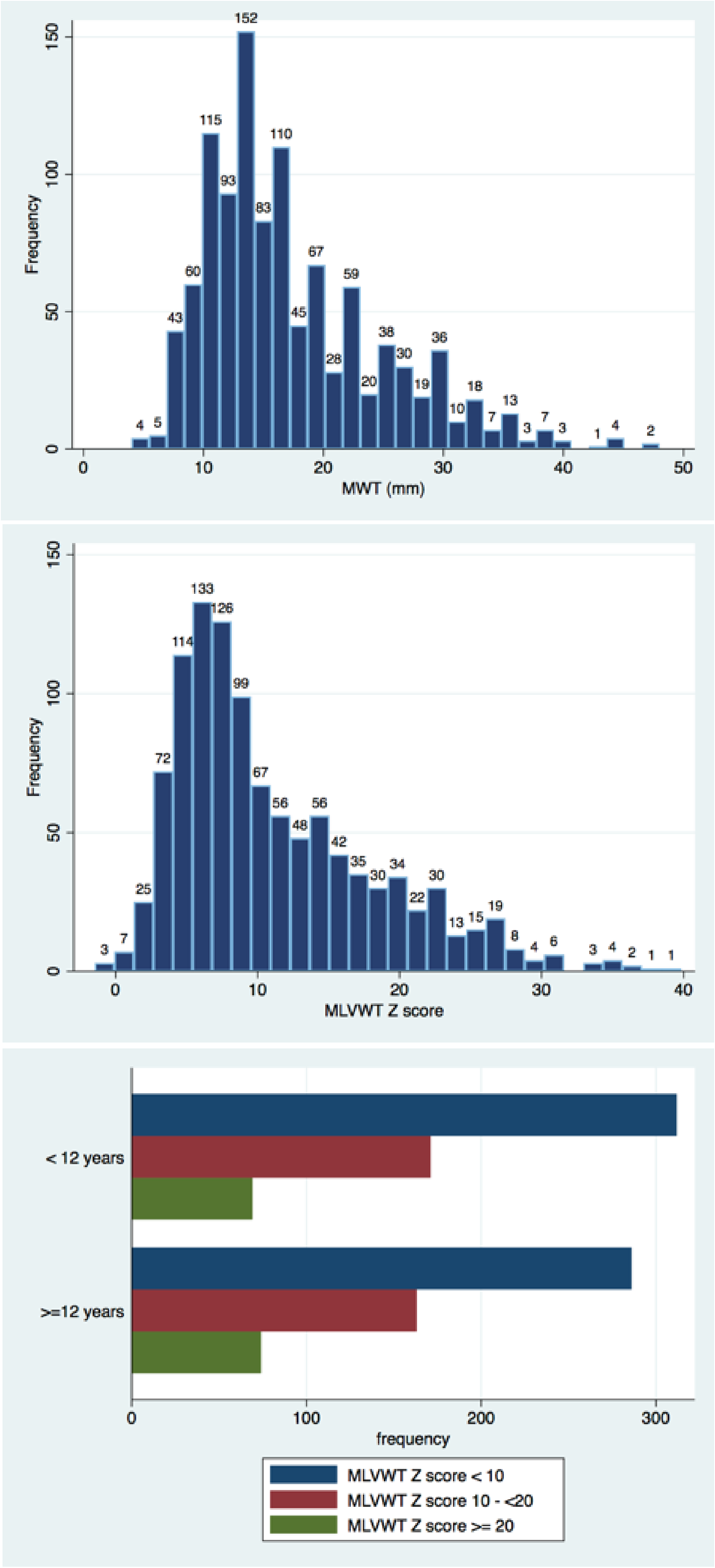

Supplementary Figure I: Distribution of left ventricular hypertrophy

a) Maximal LV wall thickness (mm)
b) Maximal LV wall thickness z score
c) Severity of hypertrophy by age of presentation
